# A systematic review of interventions targeting men's alcohol use and family relationships in low- and middle-income countries

**DOI:** 10.1017/gmh.2017.32

**Published:** 2018-03-07

**Authors:** Ali Giusto, Eve Puffer

**Affiliations:** Department of Psychology and Neuroscience, Duke Global Health Institute, Duke University, 417 Chapel Drive, Durham, NC 27708, USA

**Keywords:** Alcohol, family, interventions, low and middle-income countries

## Abstract

**Background.:**

Problem drinking accounts for 9.6% of disability-adjusted life years worldwide. It disproportionally affects men and has disabling physical, psychological, and behavioral consequences. These can lead to a cascade of negative effects on men's families, with documented ties to intimate partner violence (IPV) and child maltreatment. These multi-level problems are often exacerbated where poverty rates are high, including low and middle-income countries (LMICs). In contexts where strong patriarchal norms place men in positions of power, family-level consequences are often even more pronounced.

**Methods.:**

We conducted a systematic review of the literature on interventions in LMICs targeting men's problem drinking and any family-related outcomes. Cochrane and PRISMA procedures guided the review. The search was conducted in PsychInfo, PubMed, and Web of Science.

**Results.:**

The search yielded 1357 publications. Nine studies from four different countries met inclusion criteria. Of those, only one had the primary goal of simultaneously improving drinking and a related family-level outcome (IPV). Six of the studies documented modest improvements on both drinking and couples or family outcomes. Strategies common to these included cognitive-behavioral techniques, communication skills training, narrative therapy, and participatory learning. Gender-transformative approaches were associated with reduced IPV and more equitable gender norms, and motivational interviewing and behavioral approaches were beneficial for reducing alcohol use.

**Conclusions.:**

Findings highlight the scarcity of interventions addressing men's drinking and its effects on families, particularly for parent-child outcomes. However, results point to strategies that, combined with other evidence-based family interventions can guide the development and rigorous evaluation of integrated programs.

## Background

Problem drinking is a pervasive global mental health problem that accounts for 9.6% of disability-adjusted life years worldwide (Whiteford *et al.* 2013). Problem drinking, an umbrella-term encompassing varying levels of harmful alcohol patterns including dependence/abuse, disproportionally impacts men (Grittner *et al.* 2012; Probst *et al.* 2015) and has negative psychological, social, behavioral, and physical consequences (Rehm *et al.* 2009, 2010; Steel *et al.* 2014). Alcohol consumption above moderate levels and risky drinking patterns lead to a number of physical ailments, such as liver cirrhosis, heart failure, and certain cancers (Boffetta & Hashibe, 2006; Laonigro *et al.* 2009; Smyth *et al.* 2015). Psychologically, for men reporting problem drinking, co-morbid mental health issues, such as depression, anxiety, and externalizing problems, are common and often exacerbate alcohol-related consequences (Kessler *et al.* 2011; Grant *et al.* 2015).

Consequences of men's drinking often extend beyond the individual to impact their families (Solis *et al.* 2012). The ecological-transactional model is a helpful framework for examining effects of male drinking across family systems while also accounting for powerful and dynamic societal influences (Sameroff, 1975; Cicchetti & Lynch, 1993; Bronfenbrenner, 1994). This model may be especially helpful for examining and disentangling consequences of male alcohol use in low and middle-income countries (LMICs), given that it emphasizes the importance of cultural and contextual factors, specifically including economic factors, that impact the nature and manifestation of alcohol-related outcomes.

For men, drinking has a documented cascade of consequences on the family with clear links to negative child outcomes, partner relationship difficulties, and disrupted family systems (Leonard & Eiden, 2007). At the child-level, direct relationships have been found between male caregiver problem drinking and youth substance abuse, internalizing and externalizing problems, and poor health (Keller *et al.* 2009; Atilola *et al.* 2014). Within the caregiver-child subsystem, alcohol abuse influences the likelihood of child maltreatment, harsh parenting, lack of paternal sensitivity and warmth, and decreased cognitive stimulation in the home (Keller *et al.* 2009; Meinck *et al.* 2015). Deficits in parenting strategies may be due in part to the impact of drinking on the father's mental health, including poor emotion regulation or blunted affect, psychosocial stressors associated with drinking, and preoccupation with drug-seeking behavior (Neger & Prinz, 2015). At the couple level, intimate partner violence (IPV), marital conflict, poor communication, and poor co-parenting are all associated with men's alcohol use as shown across both high-income countries (HICs) and LMICs (Jewkes *et al.* 2010; Garcia-Moreno & Watts, 2011; Miller *et al.* 2014). Across the family system, longitudinal pathways have been documented in HICs: paternal drinking to marital conflict to child maltreatment; and paternal drinking to IPV to children's witnessing of IPV to poor child adjustment (Leonard & Eiden, 2007).

### Importance of research in LMICs

The nature, severity, and extent of the negative effects of male alcohol use are related to the context in which they occur. Yet, since the majority of research is conducted in HICs, we know very little about the influence of broader ecological factors on alcohol use in lower resourced parts of the world. Across LMICs, the most obvious common factor is high-rates of poverty that are associated with worsened individual and family consequences of alcohol use (Grittner *et al.* 2012). Consistent with this, across LMICs complex relationships exist between alcohol use, disease burden, and economic development, such that rates of consumption across LMICs are lower when compared of HICs, but the unit of disability per liter consumed is higher in LMICs and highest among those with the fewest resources (Rehm *et al.* 2009; WHO, 2014).

Additionally, specific cultural norms related to gendered power dynamics and masculinity can wield strong influences on alcohol-related consequences within individuals and across relational systems. Patriarchal norms that place men in positions of power have been associated with higher levels of men's alcohol use and negative consequences for men, women, children, and family systems (Barker *et al.* 2007). For men, these hegemonic norms are associated with increased violence, delinquency, poorer mental health, reduced help and health-seeking behavior, and increased mortality (Garfield *et al.* 2008; Wong *et al.* 2016). At the family level, associations between IPV and men's drinking are perpetuated in patriarchal climates, worsening as inequality and hegemonic norms increase (Jewkes *et al.* 2015; Wachter *et al.* 2017). Gender inequities can further impact children directly and through IPV (Garrido *et al.* 2011), as they are associated with child maltreatment, poor/absent parent involvement, and intergenerational transmission of violence (Kato-Wallace *et al.* 2014; Guedes *et al.* 2016). In sum, considering, or even explicitly addressing cultural norms, may influence the effectiveness of interventions with particular people in particular places.

### Existing evidence-based treatments

In both HIC and LMICs effective interventions for alcohol abuse exist, such as Motivational Interviewing and pharmacological treatments (Patel *et al.* 2007). Likewise, interventions exist to address problems related to dysfunctional family systems at multiple levels. Interventions such as multi-systemic therapy, functional family therapy, brief solution-focused therapy, and emotion-focused therapy address the family system as a whole (Sexton & Datchi, 2014). At the couple's level, behavioral, systemic, experiential, and emotion-focused approaches have gained strong evidence (Gurman *et al.* 2015). Targeting parent-child relationships are multiple evidence-based interventions focused primarily on parental skills training in relationship enhancement and behavioral management (Eyberg *et al.* 2008; Webster-Stratton *et al.* 2008; Sanders, 2012; Barkley, 2013; Furlong *et al* 2012); some of these also have a growing evidence base in LMICs (Mejia *et al.* 2012; Knerr *et al.* 2013).

In HICs, there are also emerging treatments addressing both family-level relationship needs *and* drinking (Powers, *et al.* 2008; Neger & Prinz, 2015). Combined treatments include programs aimed at improving alcohol abuse outcomes through family or couples therapy (Fals-Stewart *et al.* 2004). Other programs include those that treat at the individual level but include content that also targets family-related outcomes, such as integrated IPV and alcohol use programs with individual men (Kraanen *et al.* 2013). One particularly notable intervention is Alcoholic Behavioral Couples Therapy (ABCT), which targets the intersection of alcohol-use and couple-level conflict. ABCT posits that alcohol use contributes to relationship dysfunction, and that those problems, in turn, exacerbate alcohol use, creating a persistent negative cycle (Fals-Stewart *et al.* 2004). A meta-analysis of 12 randomized trials documented effects of ABCT on alcohol consumption frequency (*d* = 0.45) and marital satisfaction (*d* = 0.51) compared with control conditions and individual cognitive behavioral therapy (CBT; Powers *et al.* 2008). ABCT has also been associated with decreases in externalizing problems among children whose fathers reduced alcohol use (Andreas & O'Farrell, 2009).

Further, some alcohol-focused interventions in HICs have begun to integrate strategies to mitigate harmful parenting practices often associated with parental alcohol-abuse (Messina *et al.* 2015; Neger & Prinz, 2015). These treatments have shown both reductions in alcohol use and improved parenting (Harnett & Dawe, 2008). As one example from the USA, ABCT combined with parenting skills was associated with improved individual alcohol misuse, systemic family relationships, and child adjustment (Lam *et al.* 2009). Another intervention combining individual CBT, couples therapy, and restorative parenting sessions targeted men's alcohol-use, IPV, and parenting in a pilot trial with positive results (Stover, 2015).

Although results are promising, research on combined alcohol use and family interventions is moving forward primarily in HICs, and the need to expand to LMICs is clear. It is important to identify intervention trials in LMIC settings in which alcohol and family outcomes have both been measured. Knowing the limited nature of that work, it is then important also to identify intervention trials in LMIC settings in which these have been assessed even as secondary outcomes. Examining that literature may uncover that some of the evidence-based behavior change intervention strategies already adapted for use in LMICs may improve alcohol and family outcomes even if alcohol and family behavior changes are not the primary behavioral targets. Given the overlap between behavioral intervention strategies for a wide array of behaviors, it is likely that multiple behaviors may change at once despite a focus on specific content.

One reason that this strategy for literature review is important before deciding whether to replicate programs from HICs is the need for cultural and contextual adaptations for LMICs that may already have been done successfully for interventions being implemented in these contexts. The process of cultural adaptation – modifying interventions to address issues in contextually-relevant and meaningful ways to increase treatment viability – can range from surface level modifications to deep adaptation with the ultimate goal of increasing treatment effectiveness (Bernal *et al.* 2009; Barrera *et al.* 2013). According to the framework proposed by Bernal *et al.* (1995), adaptations can fall across the following domains: language, persons, metaphors, content, concepts, goals, methods, and context. Though there is a debate in the field regarding the necessary level of adaptation, there seems to be a consensus that some level of adaptation beyond translation is associated with more positive outcomes (Barrera *et al.* 2013; Chowdhary *et al.* 2014).

### Aims

In this paper, we systematically review interventions conducted in LMICs that measured both men's alcohol use and at least one family outcome as either primary or secondary to identify intervention strategies implemented in LMICs associated with changes in these domains. We then explore common characteristics among interventions that improved male drinking and relationship-based family outcomes and describe the strategies and implementation methods. Lastly, we aim to identify limitations in the literature and opportunities for future clinical research.

## Methods

### Inclusion criteria

Studies were included if they met the following criteria:

1. Described any intervention evaluation examining at least one alcohol-use outcome for men and at least one family-related outcome; family-related was defined as any relationship-based family variable (e.g., parenting, IPV, communication, family functioning).
2. Evaluated an intervention implemented in a LMIC, as defined by the World Bank (The World Bank Group, 2016),
3. Included a pre- and post-quantitative assessment of outcomes.

### Exclusion criteria

Studies with only women were excluded; studies with a sample that included male participants all younger than 18 or all older than 65 were also excluded. Additionally, unpublished studies, studies unavailable in English, qualitative studies, and those not published in a peer-reviewed journal were excluded.

### Search and data abstraction

Studies were identified by searching electronic databases and scanning the references of key reviews (e.g., Patel *et al.* 2007; Mejia *et al.* 2012; Panter-Brick *et al.* 2014). PsycInfo, PubMed, and Web of Science were searched with no time period limits.

Standardized search terms were applied in a sequential, stepped approach. Syntax consisted of terms and key words related to the following constructs: (a) alcohol, (b) each LMIC and setting type (e.g., ‘developing country’, ‘Uganda’), (c) intervention, (d) male inclusion, and (e) family-related (e.g., ‘father’, ‘marriage’, ‘parenting’). English language filters were applied to PubMed and Web of Science searches. See appendix for full list of search terms.

All resulting titles and abstracts were compiled and considered. The lead author (AG) assessed eligibility based on the pre-determined criteria. For the remaining articles, the full texts were reviewed, assessed for inclusion, and recorded in a database developed based on the Cochrane Consumers and Communication Review Group's data extraction template, PRISMA guidelines, and study aims (Moher *et al.* 2015). In cases where the eligibility was unclear, the first and second author discussed and reached consensus. The following information was extracted: author, year, title, city/country, study details (e.g., aims, design), participant details (e.g., age, sample size), intervention details (e.g., targets, strategies, implementation methods, context-specific adaptations), and results.

### Risk of bias

Risk of bias was coded based on adapted Cochrane Consumers and Communication Review Group guidelines (Ryan, 2013). Articles were rated on 11 criteria for the following: randomization, allocation concealment, baseline characteristic reporting, blinding, attrition, selective reporting, missing data analysis, and adequate sample size. Criteria were coded 1/yes, 0/no, or unclear; each ‘point’ equated to a reduction in bias. Studies were demarcated as ‘high’ ‘low’ or ‘medium’ risk based on resulting scores (0–4 = high, 4–7 = medium, 8–11 = low). Any study lacking randomization was considered ‘high risk’ regardless of criteria score.

## Results

In this section, we first present findings related to search results, followed by trial characteristics across studies, including study location, participants, trial design, intervention characteristics, strategies, and adaptations. We then present findings related to intervention effects on key outcomes of interest followed by effects on individual outcomes of interest with the goal of synthesizing results in a way that elucidates treatment patterns when they emerge.

### Search results

**Fig. 1.**
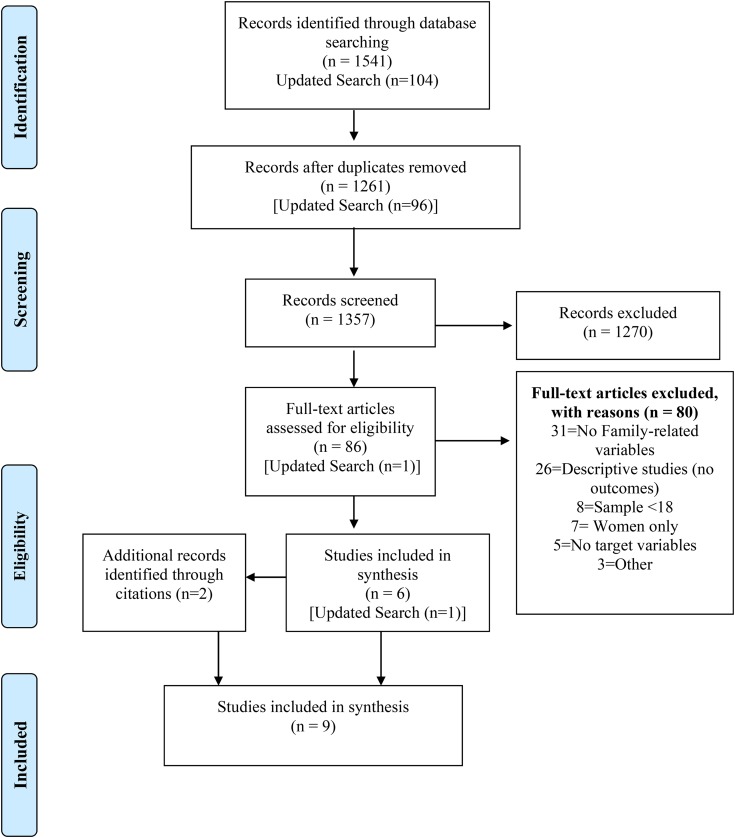
Search flow diagram.

Initial searches (19 December 2015) yielded 1541 records, and 1261 remained after removing duplicates. After the screening of titles and abstracts, 86 were eligible for full review (See Fig. 1). Full texts were then reviewed, and 80 did not meet inclusion criteria. Examining the reference lists from the remaining six articles identified two additional studies (Kalichman *et al.* 2009; Saggurti *et al.* 2013). On 23 December 2016, the search was updated, yielding 96 additional titles; these were reviewed following the same procedures and yielded one additional record (Satyanarayana *et al.* 2016). Thus, nine studies were included in this review.

### Trial characteristics

#### Location/setting

As presented in Table 1, studies were conducted in South Africa (Jewkes *et al.* 2008, 2014; Kalichman *et al.* 2009), Zambia (Jones *et al.* 2014), India (Nattala *et al.* 2010; Schensul *et al.* 2010; Saggurti *et al.* 2013; Satyanarayana *et al.* 2016), and Iran (Abdollahnejad, 2008). Trial settings included health clinics (Jones *et al.* 2014), primary care facilities (Saggurti *et al.* 2013), residential or inpatient treatment centers (Abdollahnejad, 2008; Nattala *et al.* 2010; Satyanarayana *et al.* 2016), rural communities (Jewkes *et al.* 2008), an urban slum area (Schensul *et al.* 2010), an informal settlement (Jewkes *et al.* 2014), and an urban community (Kalichman *et al.* 2009).

**Table 1.** Characteristics of included studies

Study	Country	Program	Primary aims	Design	Participants	Sample	Mean age (SD)	% Male	Outcomes (Primary = *italicized;* Secondary = plain text)	Significant findings
Jones *et al.* (2014)	Zambia	The Partner Project	Implemented and compared the effectiveness of a program to decrease high-risk sexual behavior, alcohol use, and intimate partner violence among Zambian couples affected by HIV led by professionals *v.* lay-workers	QE; treatment sequentially randomized to six selected CHC 3 Arms: 1. Waitlist control, 2. Professionally-led (RES) intervention, 3. CHC worker-led intervention Data collected at BL, 6 mo, 12 mo	Seroconcordant & serodisconcordant couples (over 18 years old) who have been together for >6 mos and sexually active in the past 30 days	394 (197 couples)	39 (8)	50%*	*Alcohol:* 5-item survey type & frequency *Family-related:* Violence [M,F] Extreme Violence [M,F] *Other Outcome:* *Sex Risk: Condom Use*	Not reduced Reduced due to time Not reduced *RES: improved (6 mo), not 12* *CHC: improved (12 mo)* *RES v. CHC: no difference.*
Kalichman *et al.* (2009)	South Africa	1.GBV/HIV intervention 2.ALC/HIV intervention	Compared the effectiveness of a gender-based, HIV prevention program to a brief alcohol and HIV intervention targeting sexual risk behavior and IPV among South African men	QE; treatment randomly assigned to 2 matched communities 2 Arms: 1. GBV/HIV 2. ALC/HIV Data collected at BL, 1 mo, 3 mo, 6 mo	Men living in two townships	475	30.2 (9.5)	100%	*Alcohol:* Alcohol before sex *Family-related:* *IPV, hit/push* [M] *Lost temper with woman* [M] *Sex Communication* *Acceptance of violence* [M] *Other outcome:* *HIV test (Y/N)* *Unprotected sex*	ALC/HIV> GBV/HIV (1 mo) *GBV/HIV>ALC/HIV (1&6 mo)* *GBV/HIV>ALC/HIV (1&6 mo)* *GBV/HIV>ALC/HIV (1 mo)* *GBV/HIV>ALC/HIV (1 mo)* *GBV/HIV>ALC/HIV (1&3 mo)* *ALC/HIV> GBV/HIV(1&3 mo)*
Jewkes *et al.* (2008)	South Africa	Stepping Stones 2nd Ed.: HIV prevention program	Evaluated the effectiveness of a behavioral HIV prevention to decrease the incidence of HIV and HSV-2 through improved gender equity and communication among South African men and women	Cluster randomized design 2 Arms: 1. Stepping Stones (SS) 2. Workshop control (C; 3 hr HIV health education) Data collected at BL, 12 mo, 24 mo	Men and women (12–23 years old) who understood consent	1360 (70 clusters)	NR (*range*:16–26)	51%	*Alcohol:* Problem drinking, AUDIT (Y/N) *Family-related:* Impregnated woman [M,F] Rape/attempted rape [M,F] Sexual/physical IPV [M,F] *Other outcome:* *Incidence of HIV* *Incidence of HSV-2*	SS>C (12 mo.) Not reduced Not reduced SS>C (24 mo.) *Not reduced* SS>C (24 mo.)
Jewkes *et al.* (2014)	South Africa	Stepping Stones 3rd Ed. & Creating Futures (SS + CF)	Piloted a combined economic empowerment -non-microloan program (CF) and an HIV-prevention program (SS) to decrease GBV, sexual risk and increase economic stability among men and women living in informal settlements	QE; Interrupted time series pilot trial 1 Arm: SS + CF Data collected at BL, BL (2 wks), 28 wks, 58 wks	Out-of-school men and women (18–34 years old)	232	NR (*range:*17–34)	47%	*Alcohol:* Problem Drinking, AUDIT *Family-related:* *Gender attitudes* [M] *Relationship control* [M,F] *Sexual/physical IPV* [M,F] *Financially support child* *Other outcome:* Condom: last sex* *Earnings* *Feelings about work* *Stolen for food*	Not reduced Improved Improved Not reduced Not improved Not improved *Improved* *Improved* *Reduced*
Schensul *et al.* (2010)	India	Research and Intervention in Sexual Health; Theory to Action (RISHTA): *Community-level arm*	Evaluated the effectiveness of a community-level STI and alcohol prevention program to decrease sexual health risk, IPV, and alcohol intake among married men from urban community slums in India	QE; two independent pre- post cross-sectional surveys conducted (CSC), plus a longitudinal sample subset followed from pre to post (LP) 1 Arm: All communities received intervention Data collected Pre-Intervention, 3 yr Post	Married men (21–40 years old) from slum communities	CSC: Survey1–2600 Survey2–2722 LP: 403	NR	100%	*Alcohol:* Never Monthly Daily *Overall drinking* *Family-related:* Spousal abuse Extramarital sex Self-assessment as husband *Other outcome:* *Masculinity scale* [M] *Sexual Performance* [M]	Improved (CSC) Reduced (CSC) Not reduced (CSC) *Not reduced (LP* *+* *CSC)* Not reduced (CSC) Not reduced (CSC) Not reduced (CSC) *Not reduced (CSC)* *Not reduced (CSC*)
Saggurti *et al.* (2013)	India	Research and Intervention in Sexual Health; Theory to Action (RISHTA): A Brief Narrative Intervention (*Healthcare level arm)*	Examined the comparative effectiveness of a brief narrative intervention across trained and untrained providers in reducing the incidence of *gupt rog,* improving sexual health, drinking, and marital behaviors among married men attending either public(BMT) or private(AYUSH) healthcare centers in 3 Indian slum communities	QE; 2 (private/public center)X2 (trained/untrained provider) design; 3 communities randomly assigned arms 3 Arms: 1. Ayurveda, Yoga, Unani, Siddha, Homeopathy (AYUSH)-private: *trained v. untrained* 2. Male Health Clinics (MHC) at primary health center-public: *trained v. untrained* 3. Control community Data collected at BL, 2–3 days, 6 mos.	Married men (21–40 years old) who seek care at experimental or control health clinics, reported a *gupt rog* (‘secret illness’) symptom, and have lived in community >1 yr.	736	30.9 (5.3)	100%	*Alcohol:* Any alcohol use (Y/N) *Family-related :* *Extramarital affairs* [M] Spousal communication [M] Self-assessment as husband [M] Intimate sexual acts with partner [M] Self-assessment as sex-partner [M] *Other outcome:* *Gupt rog consequences* *Gupt rog causes*	Time; BMT > All: reduced *Reduced (Trained)* Improved (*Trained*) No change Improved (Private & trained) No change *Reduced (Trained)* *Reduced (Trained)*
Nattala, *et al.* (2010)	India	1. Dyadic Relapse Prevention (DRP) 2. Individual Relapse Prevention (IRP)	Tested the comparative effectiveness of a relapse prevention program that included or excluded a family member in treatment and a control condition targeting alcohol use and alcohol-related dysfunction in alcohol dependent men admitted to an inpatient hospital in Bangalore, India	QE; pre-post; random treatment assigned 3 Arms: 1. DRP 2. IRP 3. Treatment as usual (TAU) Data collected at BL, 1, 2, 3, 4, 5, 6 mos.	Alcohol dependent (ICD-10) and literate men aged 20–60 who have 1 family member living with him and no co-morbid diagnoses	87	39 (8)	100%	*Alcohol:* *Days drinking* *Alcohol quantity* *Family-related:* Days without family dysfunction [M, F]	*DRP>TAU;DRP>IRP (6 mo.)* *DRP>TAU;DRP>IRP (6 mo.)* DRP>TAU;DRP>IRP (6 mo.)
Abdollahnejad (2008)	Iran	Tehran Therapeutic Community (TTC)	Evaluated pre to post outcomes of drug and alcohol use and alcohol-related dysfunction among drug & alcohol abusing men who attended an existing residential living community in Tehran	QE; pre-post 1 Arm: TCC Data collected at BL, post, 3 yrs.	Male drug & alcohol users who completed treatment at TTC	43	NR	100%	*Alcohol:* *Drug & alcohol use* *Family-related:* Social & family relationship quality [M] *Other outcome:* Psychiatric status Legal status Employment status	*Improved* Improved Improved Not improved Not improved
Satyanarayana *et al.* (2016)	India	Integrated Cognitive Behavioral Therapy (ICBI)	Evaluated the effectiveness of an 8-session cognitive behavioral treatment addressing IPV and drinking compared to TAU among married fathers in inpatient treatment for AUD	RCT 2 Arms: 1. ICBI 2. TAU Data collected at BL, 1 mo., 3 mo.	Alcohol dependent inpatient men who screened positive for IPV, married with one child younger than 16	177	38 (6.3)	100%	*Alcohol:* *Severity of Alcohol Dependence* *Family-related:* *Spousal Abuse* [M,F] Spousal depression Spousal anxiety Spousal stress Child mental health	*IBCI* = *TAU, both improved* *IBCI>TAU (1,3 mos.)* IBCI>TAU (1,3 mos.) IBCI>TAU (1,3 mos.) IBCI>TAU (1,3 mos.) Not improved

*Note*: *italicized*, primary intervention target; >, signifies the arm to the left of ‘>’ saw significant reductions in the associated variable when compared with the other treatment arm; M, male reported (on self); F, female reported (on partner); °, all finding reported refer to statistically significant change not trends for findings of interest-significance refers to statistical significance below a 0.05 alpha; RCT, randomized control trial; QE: Quasi-experimental; grp, group; NR, not reported; IPV, intimate partner violence; BL, baseline; mo, month; wk, week; yo, year old; MHC, biomedical primary health center providers; AYUSH, Ayurveda, Yoga, Unani, Siddha, Homeopathy providers; TAU, treatment as usual; ICD-10, international classification of diseases; NGO, non-governmental organization; CSC, cross sectional surveys; LP, longitudinal panel; CHC, community health center; GBV, gender-based violence; RES, research-led; SS, stepping stones; CF, creating futures; Y/N, yes or no; *‘gupt rog’*, Indian term for sexually transmitted infections, fertility and sexual problems; AUDIT, alcohol use disorders identification test; IRP, individual relapse prevention; DRP, dyadic relapse prevention; TTC, tehran therapeutic community.

#### Participants

Participants were between the ages of 16 and 47 years with samples ranging from 43 participants (Abdollahnejad, 2008) to 2600 (Schensul *et al.* 2010; See Table 2). Three studies included both men and women; of those, one included HIV seroconcordant and serodiscordant couples (Jones *et al.* 2014), and two consisted of young men and women (Jewkes *et al.* 2008, 2014). Of the six that only included men, two were conducted with married men seeking sexually transmitted infection (STI) treatment (Schensul *et al.* 2010; Saggurti *et al.* 2013), one included married men in treatment for alcohol dependence with children (Satyanarayana *et al.* 2016), one among men in the general population (Kalichman *et al.* 2009), and two among men in treatment for alcohol dependence (Abdollahnejad, 2008; Nattala *et al.* 2010).

**Table 2.** Description of interventions

Program	Format	Amount	Theory	Core intervention strategies	Study	Quality assurance	Therapist	Training
The Partner Project	Single-sex, parallel groups; Couples homework Delivered by CHC or RES	4, 2 hr. sessions	Theory of Reasoned Action & Planned Behavior; Formative work	Psychoeducation (e.g., video FAQ) Critical discussion and feedback Behavioral skills practice (e.g., role play) CBT strategies (e.g., relaxation) HIV risk reduction strategies *Key Content:* sexual health and risk, IPV, sexual negotiation *Other:* Condoms offered	Jones *et al.* (2014)	Audio recorded session; Quality checklist with 10% sample; Manual	CHC: CHC senior staff selected ‘appropriate’ CHC group leaders RES: Project research staff (education NR)	CHC: 2-day dyadic; intervention observation; train-the-trainer RES: Partner-trained staff
1. GBV/HIV	Small single-sex group (8–12)	5, 3 hr. sessions; 1 wk	Behavioral theory; social cognitive learning; social constructivist; Formative work	Group support (e.g., songs, chants) Psychoeducation (e.g.,video testimonials) Critical discussion and feedback Problem solving Behavioral skills practice (e.g., role play) HIV risk reduction strategies Communication skills Goal-setting Advocacy training and outreach *Key Content:* sexual risk, values, masculinities, GBV consequences	Kalichman *et al.* 2009	Supervision; Manual	Male & female team; previous HIV counseling experience	Manual, weekly supervision, flip charts with steps
2. ALC/HIV	Small single-sex group (8–12)	1, 3 hr. session	*Same as Above*	Psychoeducation Critical discussion and feedback Behavioral skills practice Behavioral self-management Communication skills Motivational interviewing Goal-setting *Key Content:* alcohol, sexual risk		*Same as Above*	*Same as Above*	*Same as Above*
Stepping Stones (SS) 2nd Ed.:HIV prevention program	Single-sex, parallel groups + 4 mixed-sex groups	13, 3 hr. sessions + 3 mixed-gender group + 1 community meeting, 6–8 wks	Adult Education theory (primary); Freirian models of self reflection; Assertiveness training	Group support Critical discussion and feedback Behavioral skills practice (e.g., role play, dramas) Assertiveness training *Key Content:* Sexual/HIV and physical health, motivations, agency, love, pregnancy, GBV, gender norms *Other:* Includes refreshments	Jewkes *et al.* (2008)	Attendance; Manual	PPASA NGO staff, slightly older, education or life skills training; gender sensitive/open-minded	2 wk. training; two practice groups
Stepping Stones (SS) 3rd Ed. & Creating Futures (CF)	Single-sex, parallel groups	SS: 10, 3 hr. session, 1 mixed-gender peer session + CF: 11, 3 hr sessions *Total:*22, 3 hr sessions, 2 per wk over 12 wks	*Same as SS* CF: Theory of sustainable livelihoods and practice	*SS*: *Same as above* CF: Critical discussion and feedback Behavioral skills practice (e.g., role play, dramas) Resources strengthening skills Economic empowerment and skills Goal-setting *Key Content:* livelihood, resources, coping with crises, finances, business, past experiences/strengths, debt, community support, career expectations	Jewkes *et al.* (2014)	Attendance; ‘ad hoc’ trainer visits to session; Manual	Empower NGO staffers who completed secondary school; experience in health sector & facilitation	Training on gender equity & attitudes, HIV & AIDS, sexual & reproductive health, facilitation skills
Research and Intervention in Sexual Health: Theory to Action (RISHTA): *Community-level arm*	Community leaflets, videos, movies, discussions	*Dissemin-ated throughout community in multiple forms*	Ecological theory; Formative qualitative work	*Multi-level community components:* Street Dramas Community Meetings: discussion, Q&A Poster Sessions and Banner Presentation Videos/Movies Printed Materials Interpersonal Communication: referrals, discussion	Schensul *et al.* (2010)	Exposure to messages; Recollection of messages survey	Trained RISHTA staff	NR
RISHTA: A Brief Narrative Intervention (*Healthcare level arm)*	Individual: primary/public or holistic/private care	~1–3 sessions, 20–40 minutes	Theory-driven ecological approach; Formative qualitative work	Psychoeducation Medical Exam (e.g., STI diagnosis, medical treatment) Partner notification CBT strategies (e.g., reframing), Narrative therapy strategies (e.g., in-depth assessment, discussion of content) *Key Content:* sexual health, *gupt rog* symptoms, etiology, consequences, prior treatment seeking, masculinity, marital relationship	Saggurti *et al.* (2013)	Recollection of treatment; Manual	1. AYUSH provider + narrative training; 2. MHC biomendical providers + narrative training; 3. AYUSH or MHC provider with no narrative training	Providers trained in Narrative Intervention by key experts from Population Sciences –Mumbai & The University of Connecticut School of Medicine for 16 hrs., 4 days + 9, 1–2 hr. session over 2 yrs.
1. DRP 2. IRP	DRP: dyadic inpatient therapy + 1 month follow-up IRP: individual inpatient therapy + 1 mo. follow-up TAU: individual inpatient	DRP & IRP: 8–10, 2–3 per wk., ~1 hr. 4 wks. TAU: varied	Behavioral theory, Family systems, Family disease approach	*DRP*: Critical discussion and feedback Dyadic behavioral skills practice Behavioral management (e.g., scheduling) Defining family roles Refusal skills Problem solving Family abstinence contract *Key Content:* family support, alcohol triggers and impact, financial management *IRP*: *Same as DRP* minus family involvement and defining family roles. *TAU*: Psychoeducation Detox, long-term medication Discussion and feedback Refusal skills *Key Content:* managing triggers, abstinence advice, positive lifestyle	Nattala *et al.* (2010)	Sessions observed by center psychiatrist; Attendance; Manual	DRP & IRP delivered by 1^st^ author (psychiatric nurse)	Author trained at hospital >1 yr.
Tehran Therapeutic Community	Residential living community	6-month, residential treatment	NR	Structured employment hierarchy Behavioral management (e.g., strict schedule) Daily recreation activities Group therapies (e.g., CBT, music, family) Vocational counseling	Abdollahnejad (2008)	NR	NR	NR
Integrated Cognitive Behavioral Therapy (ICBI)	ICBI: Individual, inpatient TAU: Individual	ICBI: 8 sessions, 45–60 minutes [+TAU] TAU: 1 session; medication	Cognitive- behavior therapy	*ICBI*: Psychoeducation CBT strategies (e.g., cognitive restructuring; identifying triggers) Anger management Assertiveness Training Relaxation [Medication management] *Key Content:* IPV and alcohol use -relationship, triggers, consequences TAU: Psychoeducation (alcohol) Medication Management	Satyanarayana *et al.* (2016)	Session audio tapes reviewed for treatment adherence & fidelity by clinical psychologist	Masters in psychology	Certificate course in ICBI; additional training from lead author

*Note*: IPV, intimate partner violence; GBV, gender-based violence; SS, stepping stones; CF, creating futures; RES, research-led; CHC, community health center; DRP, dyadic relapse prevention; IRP, individual relapse prevention; TAU, treatment as usual; NR, not reported; FAQ, frequently asked questions; CSC, cross-sectional survey; CBT, cognitive-behavioral therapy; Q&A, question and answer; AUD, alcohol use disorder; formative work, in-country work or adaptation completed prior to intervention implementation; NGO, non-governmental organization; MHC, biomedical primary health center providers; AYUSH, Ayurveda, Yoga, Unani, Siddha, Homeopathy providers; PPASA, Planned Parenthood Association of South Africa; hrs., hours; yrs., years; wk.=week.

#### Design

The majority of trials employed quasi-experimental designs with two randomized control trials (RCTs) (Jewkes *et al.* 2008; Satyanarayana *et al.* 2016; See Table 1). Six of the nine studies compared the primary intervention to a comparison group (Jewkes *et al.* 2008; Kalichman *et al.* 2009; Nattala *et al.* 2010; Saggurti *et al.* 2013; Jones, *et al.* 2014; Satyanarayana *et al.* 2016). Of these, comparison groups consisted of different provider type (Saggurti *et al.* 2013; Jones *et al.* 2014), treatment as usual (Satyanarayana *et al.* 2016), a psychoeducation workshop (Jewkes *et al.* 2008), a different delivery format – individual *v.* family (Nattala *et al.* 2010), and an active treatment condition (Kalichman *et al.* 2009). Of the remaining three studies, one employed a pre-post interrupted time-series design (Jewkes *et al.* 2014), and two employed a pre-post design with no comparison groups (Abdollahnejad, 2008; Schensul *et al.* 2010).

#### Intervention characteristics

Within the nine studies, ten interventions were evaluated because one study (Kalichman *et al.* 2009) included a comparison intervention active enough and dissimilar enough from the tested intervention to be examined. Intervention aims were diverse. Only one intervention had the primary aim to target both alcohol use and IPV among men (Satyanarayana *et al.* 2016); rather, the most common primary intervention targets were sexual risk and STIs (Jewkes *et al.* 2008; Kalichman *et al.* 2009; Saggurti *et al.* 2013; Jones, *et al.* 2014), followed by alcohol-use (Abdollahnejad, 2008; Nattala *et al.* 2010; Schensul *et al.* 2010). One trial targeted both gender-based violence (GBV) and sexual risk behavior as primary outcomes (Kalichman *et al.* 2009), while another targeted both GBV and financial earnings (Jewkes *et al.* 2014). Interventions were delivered by a range of professionals and non-specialists (Table 2).

##### Intervention strategies

Table 2 describes strategies implemented across interventions and theoretical underpinnings. Every intervention employed elements of structured discussion, goal-directed feedback (e.g., alternative suggestions), and psychoeducation targeting unique aims. Seven of ten programs stated use of participatory learning techniques such as group discussion, role-play (Kalichman *et al.* 2009; Nattala *et al.* 2010; Jones *et al.* 2014; Satyanarayana *et al.* 2016), and dramas (Jewkes *et al.* 2008, 2014). Communication skills were taught in five programs that each included a focus on GBV/IPV (Jewkes *et al.* 2008; Kalichman *et al.* 2009; Jewkes *et al.* 2014; Jones *et al.* 2014). Three used gender-transformative approaches for addressing GBV/IPV (Jewkes *et al.* 2008, 2014; Kalichman *et al.* 2009). Cognitive-behavioral strategies were also described in six of ten programs (Abdollahnejad, 2008; Kalichman *et al.* 2009; Saggurti *et al.* 2013; Jones *et al.* 2014; Satyanarayana *et al.* 2016); of these, one was delivered as individual therapy (Saggurti, *et al.* 2013).

Unique strategies also emerged with three programs specifically employing assertiveness techniques (Jewkes *et al.* 2008, 2014; Satyanarayana *et al.* 2016) and one teaching alcohol refusal skills (Nattala *et al.* 2010). One program helped strengthen job skills (Jewkes *et al.* 2014), and another taught financial budgeting (Nattala *et al.* 2010). Only one used motivational interviewing for alcohol use (Kalichman *et al.* 2009); one used narrative techniques (Saggurti *et al.* 2013); and one explicitly targeted the link between alcohol use and IPV using CBT principles (Satyanarayana *et al.* 2016). Lastly, a community-level program applied many strategies to increase community awareness and education (Schensul *et al.* 2010).

##### Intervention adaptations

Intervention adaptations ranged from surface level modifications (i.e., basic translation) to deep adaptation (i.e., modified rationale and intervention strategies) to the development of a new intervention for the context (Table 3). Six studies applied deep adaptation to previous interventions based on formative community-based work, such as interviews and focus groups, piloting, and community partnerships (Kalichman *et al.* 2009; Schensul *et al.* 2010; Saggurti, *et al.* 2013; Jones *et al.* 2014; Satyanarayana *et al.* 2016). Three interventions were considered to have surface-level adaptations, such as translations or changes to the structure of the intervention that did not significantly change the content or strategies (Jewkes *et al.* 2008, 2014; Nattala *et al.* 2010). Lastly, a residential program was not adapted *per se*, as it was implemented based on general therapeutic community principles (Abdollahnejad, 2008).

**Table 3.** Contextual and cultural considerations of reviewed interventions

Study	Program	Country	Setting	Rationale for the intervention	Adaptation Level	Formative Work: Y/N	Notes on adaptation
Jones *et al.* (2014)	The Partner Project	Zambia	Six urban Community Health Clinic	High rates of HIV Limited human resources: Need for community-based intervention	L; DA of PTT; PF	Yes^a^^,^^b^	Years of formative research from 1999 on (e.g., multiple previous trials in Zambia) Partnerships with government community advisory boards
Kalichman *et al.* (2009)	1. GBV/HIV 2. ALC/HIV	South Africa	Community Health Clinic	High-rates of HIV driven by men High-rates of GBV driven by men	L; DA; Developed-for-setting PTT	Yes^c^	In-depth focus groups: Men & women from informal settlements Workshops: Collaborators & experts Previous pilot testing
Jewkes *et al.* (2008)	Stepping Stones 2nd Ed.:HIV prevention program	South Africa	Rural communities Eastern Cape province	High-rates of HIV High-rates of GBV	L; SA; PTT	NI^#^	Translation and structural surface changes based on setting *Original theory proposed for use in Uganda appears unchanged*^#^
Jewkes *et al.* (2014)	Stepping Stones 3rd Ed. & Creating Futures	South Africa	Informal settlements	High-rates of HIV High-rates of GBV High-rate of poverty Limited human resources: Need for community-based intervention	L; SA; Integration of PTTs	NI^#^	Translation and structural surface changes based on setting (e.g., excluding peer advocacy component due to financial constraints) Original theory proposed for use in Uganda appears unchanged^#^
Schensul *et al.* (2010)	RISHTA: *Community-level arm*	India	Three slum communities outside of Mumbai	High-rates of STIs High-rate of poverty	Developed-for-setting	Yes	Qualitative interviews: Community-members & key stakeholders
Saggurti *et al.* (2013)	RISHTA: *Healthcare-level arm*	India	1. AYUSHA Private Healthcare Centers 2.Public Male Health Clinic (MHC) biomedical care center	High-rates of STIs High-rate of poverty Limited human resources: need for task-shifting	DA; Developed-for-setting	Yes	Qualitative interviews: Community-members & key stakeholders
Nattala *et al.* (2010)	DRP & IRP	India	National Hospital Deaddiction Center (inpatient)	High rate of alcohol relapse	L; Integrated; CV	Partial	Integrated existing manuals from USA and India Content translated Expert review: hospital psychiatrists
Abdollahnejad (2008)	Tehran Therapeutic Community	Iran	Residential therapeutic community	High rate of substance use	Already Existing	No	Existing treatment
Satyanarayana *et al.* (2016)	ICBI	India	Inpatient hospital	High rates of co-morbid alcohol use and IPV	PTT; DA; Developed for setting	Yes^d^	Formative in-depth interviews: married heavy drinking men who reported to have perpetrated IPV (*N* = 10) & their spouses (*N* = 10)

*Note*: NI, not enough information to make a determination; #, for this intervention, there is an adapted version of the original manual for South Africa^e^, but the methods for adaptation are not published to our knowledge and as such information from published material is presented, but may miss important aspects of adaptation; CHC, community health clinic; PTT, previously tested treatment; DA, deep adaptation; SA, surface adaptation; CV, content validation; PF, participatory feedback; W, workshop; L, language; UC, unclear; RES, research-led; SSA, sub-Saharan Africa; SA, South Africa; DRP, dyadic relapse prevention; IRP, individual relapse prevention; RISHTA, research and intervention in sexual health: theory to action; NGO, non-governmental organization; STI, sexually transmitted infection; *, time and frequency not reported; NR, not reported; MHC, biomedical primary health center; AYUSH, Ayurveda, Yoga, Unani, Siddha, Homeopathy providers; GBV, gender based violence; ICBI, integrated cognitive behavioral therapy.

a Jones DL, Weiss SM, Chitalu N, Villar O, Kumar M, Bwalya V, Mumbi M (2007) Sexual risk intervention in multiethnic drug and alcohol users. American Journal of Infectious Diseases 3(4), 169.

b Jones D, Kashy D, Chitalu N, Kankasa C, Mumbi M, Cook R, Weiss S (2014) Risk reduction among HIV-seroconcordant and-discordant couples: the Zambia NOW2 intervention. *AIDS patient care and STDs* 28(8), 433–441.

c Simbayi LC, Cloete A, Strebel A, Henda N, Kalichman SC, Cherry C, Kalichman M, Crawford M, Cain D, Shefer T, Thabalala M (2008) HIV/AIDS risk reduction and domestic violence intervention for South African men: theoretical foundations, development, and test of concept. International Journal of Men's Health 7, 254–272.

d Satyanarayana VA, Hebbani S, Hegde S, Krishnan S, Srinivasan K (2015). Two sides of a coin: Perpetrators and survivors perspectives on the triad of alcohol, intimate partner violence and mental health in South India. *Asian Journal of Psychiatry* 15, 38–43.

e Jewkes R, Nduna M, Jama PN (2002) Stepping Stones, South African Adaptation, 2nd edn. Medical Research Council, Pretoria, South Africa.

#### Outcome measures

One intervention targeted alcohol use *and* a family-related variable as the primary specified outcomes of change (Satyanarayana *et al.* 2016; See Table 1). As described, sexual risk/STI reduction was the most common primary target. Measures of alcohol-use included frequency and quantity of use (Nattala *et al.* 2010; Schensul *et al.* 2010; Jones *et al.* 2014), drug *and* alcohol consumption (Abdollahnejad, 2008), any alcohol use (Yes/No; Saggurti *et al.* 2013), alcohol use before sex (Kalichman *et al.* 2009), severity of alcohol dependence (Satyanarayana *et al.* 2016), and problem drinking (Yes/No; Jewkes *et al.* 2008, 2014). Time frames ranged from 12 months (Jewkes *et al.* 2014) to within the past week (Jones *et al.* 2014). For family-related variables, IPV/GBV were most commonly measured (Jewkes *et al.* 2008, 2014; Kalichman *et al.* 2009; Schensul *et al.* 2010; Saggurti *et al.* 2013; Jones *et al.* 2014; Satyanarayana *et al.* 2016); three of these identified IPV/GBV as a primary target (Kalichman *et al.* 2009; Jewkes *et al.* 2014; Satyanarayana *et al.* 2016). Three studies included other family-level variables as secondary outcomes including days without family dysfunction (Nattala *et al.* 2010); quality of family and social relationships (Abdollahnejad, 2008); and spousal stress, anxiety, depression, and child mental health (Satyanarayana *et al.* 2016).

#### Interventions impacting alcohol use *and* family-related outcomes

Table 1 presents each trial's results for the outcomes of interest. Although only one intervention had the primary aim of targeting both alcohol-use and a family-related outcome, results indicated that five interventions were associated with improvements in both alcohol use and at least one family-related outcome (Abdollahnejad, 2008; Jewkes *et al.* 2008; Nattala *et al.* 2010; Saggurti *et al.* 2013; Satyanarayana *et al.* 2016). One RCT comparing treatment as usual and a CBT intervention showed decreased severity of alcohol dependence across both conditions, while the CBT group showed greater improvements in IPV and secondary outcomes of spousal depression, stress, and anxiety; no improvements in child well-being were detected (Satyanarayana *et al.* 2016). Next, the RCT of an HIV and STI risk intervention, ‘Stepping Stones’, showed improvements in secondary outcomes of problem drinking and sexual/physical violence; primary outcome results showed reduced herpes simplex virus (HSV-2) but no significant effects on HIV incidence (Jewkes *et al.* 2008). The Dyadic Relapse Prevention (DRP) program showed pre-post reductions in the primary alcohol use outcome and secondary outcome of family functioning (Nattala *et al.* 2010). A brief narrative intervention achieved primary outcomes of reducing ‘*gupta rog’* symptoms, a catchall Indian term for STIs and sexual problems, and decreased secondary outcomes of reduced alcohol use, reduced extramarital affairs, improved spousal communication, and more equitable gender attitudes (Saggurti *et al.* 2013). The Tehran residential therapeutic community (TCC) showed significant improvements in drug and alcohol use and secondary outcome improvements of social and family relationships (Abdollahnejad, 2008).

### Interventions impacting alcohol use *or* family-related outcomes

Three interventions showed either drinking *or* family outcome improvements. The study by Kalichman *et al.* (2009) comparing the effectiveness of two interventions – (1) an integrated GBV and HIV prevention program (GBV/HIV) and (2) a briefer alcohol and HIV prevention program (ALC/HIV) on sexual risk and GBV perpetration – found the interventions led to different improvements (Kalichman *et al.* 2009). ALC/HIV was associated with improvements in secondary outcomes of alcohol use before sex but not GBV. Conversely, the GBV/HIV intervention did not reduce alcohol use but was associated with improvements in the primary outcome of GBV, loss of temper with a woman, sexual communication, and acceptance of violence (Kalichman *et al.* 2009). Next, ‘Stepping Stones’, the HIV prevention program, was combined with a financial strengthening intervention and showed improved secondary couple-level variables of men's gender attitudes and relationship control (Jewkes *et al.* 2014). Unlike the previous RCT evaluation of ‘Stepping Stones’ alone (Jewkes *et al.* 2008), problem drinking did not significantly decrease for men, but mental health improved and women reported decreased physical/sexual violence.

### Risk of bias

Two studies were randomized and analyzed at the level of randomization with a low risk of bias (Jewkes *et al.* 2008; Satyanarayana *et al.* 2016), while the remaining seven were considered high-risk given a lack of randomization. High-risk of bias invites caution when interpreting results across findings. However, for the non-randomized studies, it should be noted that the methodologies typically matched study purpose (e.g., interrupted time series for pilot/feasibility purposes).

## Discussion

The intersections between men's problem drinking and family consequences present a unique opportunity for combined interventions targeting improvements on multiple outcomes from alcohol use to child mental health, family functioning, violence, and intergenerational cycles of risk. The purpose of this review was to examine the extant literature on interventions targeting both alcohol and family-related outcomes with men in LMICs. In total, nine studies and ten interventions met inclusion criteria. Of those, one had the primary goal of improving both drinking *and* related family-level outcomes (Satyanarayana *et al.* 2016); the remainder included one (two studies) or both (seven studies) as secondary, with many focusing primarily on sexual risk. Most family outcomes related to couples well-being, and no studies targeted parent-child relationships.

Despite the lack of direct focus on alcohol and family outcomes, over half of the studies documented modest improvements in both outcomes. Additionally, despite heterogeneity across studies, results point to promising core intervention strategies. Here, we discuss these strategies and the ways that future interventions can build on them by combining results with broader evidence on family-based interventions. Given the lack of interventions targeting alcohol and parent-child outcomes, we include how lessons learned from this review may apply to possibilities for combining alcohol- and parenting-focused strategies.

### Intervention strategies

Interventions that improved both drinking and family outcomes included cognitive and behavioral strategies, communication skills training, and narrative techniques often taught through participatory learning. What these approaches have in common is that they are grounded in previous evidence and have been applied to changing a wide range of behaviors. It follows that having such strategies at the core of future combined interventions would allow participants to learn skills that they can then apply to both drinking and relationship goals. For instance, problem-solving can be applied to identify consequences and find alternatives to both drinking and IPV. Further, aiming to target these outcomes directly is likely important for specifically improving them (Satyanarayana *et al.* 2016). Incorporating family members or partners in treatment also emerged as a potentially important element for seeing multi-target improvements (Nattala *et al.* 2010; Jewkes *et al.* 2014). This complements HIC literature showing couples/family treatments typically outperform individual approaches for addressing alcohol use, couple conflict, and mental health (Baucom *et al.* 2012). Yet, family member inclusion may not always be necessary to see improvements in family-level outcomes (Satyanarayana *et al.* 2016).

Effective strategies also emerged that were specific only to one outcome. For alcohol use, these included motivational interviewing (MI), behavioral management, and goal-setting (Jewkes *et al.* 2008; Kalichman *et al.* 2009; Nattala *et al.* 2010), which are consistent with the larger evidence-base (Benegal *et al.* 2009). For family outcomes, gender-transformative approaches were associated with reduced IPV (Jewkes *et al.* 2008, 2014; Kalichman *et al.* 2009), the most commonly included relationship outcome. This supports growing evidence that shifting unequal gender norms and targeting hegemonic masculinity can reduce GBV (Jewkes *et al.* 2015).

### Integrating the broader evidence base on family interventions

Given the limited targets of the interventions identified through this review, results should be examined alongside existing dual-target intervention approaches described in the introduction and the larger family intervention evidence base. Taken together, we can better discern opportunities for a broader range of combined interventions to reduce alcohol use and improve couples’ and parent–child relationships across contexts.

Outside of the literature on alcohol use, parenting intervention studies in both HICs and LMICs points to strategies to consider for alcohol-family interventions. Most clearly, behavioral parenting interventions that strengthen skills for positive interactions and effective behavior management have a strong evidence base in HICs (Kaminski *et al.* 2008). They are also gaining evidence in LMICs (see reviews by Mejia *et al.* 2012; Knerr *et al.* 2013). As examples, parenting and family programs have shown positive impacts among caregivers in Liberia (Puffer *et al.* 2015), caregivers in South Africa (Cluver *et al.* 2016), and Burmese migrant families (Puffer *et al.* in press). They have also documented effects on mental health symptoms of children (Jordans *et al.* 2013; Annan *et al.* 2016). The evidence is therefore, converging to provide the foundation for combining effective parenting intervention strategies with interventions for other outcomes, such as alcohol use, that also affect the family system. One challenge to tackle when combining interventions to target male problem drinking is that fathers have often been under-represented in parenting programs. This is in part due to difficulties engaging men in treatment – a task made more challenging by alcohol use (Cowan *et al.* 2009). As men hold responsibility and power influencing family outcomes, Panter-Brick and colleagues (2014) describe their inclusion as a potential ‘game-change’ in the field of child and family health.

Adapting existing dual-target interventions evaluated in HICs to LMICs represents another avenue for addressing alcohol use and family relationships in these settings. Given the growing evidence base for alcohol-family treatments in HICs and the successes of culturally adapted programs (Castro *et al.* 2010), it follows that adaption of evidence-based programs would be a viable option. Nattala and colleagues (2010) – an included study – further demonstrated the promise of this approach when using ABCT as one of three manuals from which they developed the Dyadic Relapse Prevention program in India with positive outcomes on intended alcohol and relationship targets (Nattala *et al.* 2010)

Efforts to combine promising strategies would be well-timed, as emerging approaches to mental health treatment have the explicit goal of combining strategies in ways that can reach multiple outcomes in a cohesive, parsimonious, and effective manner (Chorpita *et al.* 2005; Barlow *et al.* 2013; Murray *et al.* 2014). Transdiagnostic and modular strategies represent two such approaches. Transdiagnostic approaches identify and target common and core maladaptive features underlying categorized dysfunctions (e.g., depression) that are not disorder-specific (e.g., interpersonal difficulties maintaining substance use, depression, IPV); those overlapping features can then be targeted through a set of common treatment elements (Murray *et al.* 2014). Modular approaches work to integrate complementary strategies into unique intervention packages; full interventions can be sub-divided into meaningful, stand-alone components to be flexibly implemented alone or in complement (Chorpita *et al.* 2005; Lyon *et al.* 2014).

### Limitations

This review is limited by study heterogeneity and high-risk designs that precluded the ability to conduct a meta-analysis and point to the need for more rigorous evaluation designs. Variability in measurement also limited the conclusions drawn, with only two studies using the same assessment of alcohol use (Jewkes *et al.* 2008, 2014). Additionally, the search approach, while systematic, cannot guarantee the identification of all interventions as it is subject to publication and language bias. Related, it is possible some interventions were not included in the review if a secondary outcome variable of interest, such as alcohol, was not noted in their methods, abstract, keyword, or title. The search and data extraction also were primarily conducted by the first author rather than having multiple independent raters. Lastly, including unpublished and qualitative results may have identified additional intervention approaches.

## Conclusion

This systematic review identified nine peer-reviewed intervention studies conducted with men in LMICs that included measures of alcohol-use and a family-related outcome. Five interventions led to improvements in both alcohol and family outcomes. Those often used cognitive-behavioral strategies, communication skills, narrative techniques, and participatory learning approaches. Three interventions showed improvements in either an alcohol or a family related outcome using motivational interviewing and behavioral approaches, and gender-transformative strategies, respectively. Overall, results highlight the scarcity of interventions addressing men's drinking and its effects on families, particularly related to parent-child outcomes. However, results of those that do exist suggest the feasibility and likely benefits of combined approaches. Future interventions can target a broader range of family relationships that are affected by alcohol use by integrating promising strategies with other evidence-based couples and parenting interventions as well as exploring the adaptation of combined approaches effective in HICs.

## Supplementary material

For supplementary material accompanying this paper visit http://dx.doi.org/10.1017/gmh.2017.32. click here to view supplementary material
