# Supplementary material for: A systematic review of interventions targeting men's alcohol use and family relationships in low- and middle-income countries
Source: Glob Ment Health (Camb). 2018 Mar 7;5:e10. doi: 10.1017/gmh.2017.32 (PMC5885490; doi:10.1017/gmh.2017.32)
Supplement: Supplementary file 1 [file S2054425117000322sup001.docx]

**APPENDIX**

Systematic Review Search Terms and Initial Search Times*

Pubmed* Limited to Human species studies January, 21 2016 at 3:30pm

| Set # | Search Terms | # of Results |
| --- | --- | --- |
| 1 | "Alcohol-Related Disorders"[Mesh] OR "Alcohol Drinking"[Mesh] OR "Alcoholism"[Mesh] OR "drinking behavior"[MeSH Terms] OR problem drink*[tiab] OR heavy drink*[tiab] OR alcohol problem*[tiab] OR risk drink*[tiab] OR at-risk drink*[tiab] OR alcohol depend*[tiab] OR excessive drink*[tiab] OR excessive alcohol*[tiab] OR "alcohol consumption"[tiab] OR alcohol addiction*[tiab] OR risk alcohol*[tiab] OR risk drink*[tiab] OR "alcohol misuse"[tiab] OR "alcohol abuse"[tiab] OR hazardous alcohol*[tiab] OR hazardous drink*[tiab] OR harmful alcohol*[tiab] OR harmful drink*[tiab] OR (("drinking"[tiab] OR "drinkers"[tiab]) AND "alcohol"[tiab]) | 172,682 |
| 2 | "Developing Countries"[Mesh] OR "Afghanistan"[Mesh] OR "Bangladesh"[Mesh] OR "Benin"[Mesh] OR "Burkina Faso"[Mesh] OR "Burundi"[Mesh] OR "Cambodia"[Mesh] OR "Central African Republic"[Mesh] OR "Chad"[Mesh] OR "Comoros"[Mesh] OR "Democratic Republic of the Congo"[Mesh] OR "Eritrea"[Mesh] OR "Ethiopia"[Mesh] OR "Gambia"[Mesh] OR "Guinea"[Mesh] OR "Guinea-Bissau"[Mesh] OR "Haiti"[Mesh] OR "Kenya"[Mesh] OR "Democratic People's Republic of Korea"[Mesh] OR "Liberia"[Mesh] OR "Madagascar"[Mesh] OR "Malawi"[Mesh] OR "Mali"[Mesh] OR "Mozambique"[Mesh] OR "Myanmar"[Mesh] OR "Nepal"[Mesh] OR "Niger"[Mesh] OR "Rwanda"[Mesh] OR "Sierra Leone"[Mesh] OR "Somalia"[Mesh] OR "Tajikistan"[Mesh] OR "Tanzania"[Mesh] OR "Togo"[Mesh] OR "Uganda"[Mesh] OR "Zimbabwe"[Mesh] OR "Armenia"[Mesh] OR "Bhutan"[Mesh] OR "Bolivia"[Mesh] OR "Cameroon"[Mesh] OR "Cape Verde"[Mesh] OR "Congo"[Mesh] OR "Cote d'Ivoire"[Mesh] OR "Djibouti"[Mesh] OR "Egypt"[Mesh] OR "El Salvador"[Mesh] OR "Georgia (Republic)"[Mesh] OR "Ghana"[Mesh] OR "Guatemala"[Mesh] OR "Guyana"[Mesh] OR "Honduras"[Mesh] OR "Indonesia"[Mesh] OR "India"[Mesh] OR "Kosovo"[Mesh] OR "Kyrgyzstan"[Mesh] OR "Laos"[Mesh] OR "Lesotho"[Mesh] OR "Mauritania"[Mesh] OR "Micronesia"[Mesh] OR "Moldova"[Mesh] OR "Mongolia"[Mesh] OR "Morocco"[Mesh] OR "Nicaragua"[Mesh] OR "Nigeria"[Mesh] OR "Pakistan"[Mesh] OR "Papua New Guinea"[Mesh] OR "Paraguay"[Mesh] OR "Philippines"[Mesh] OR "Independent State of Samoa"[Mesh] OR "Atlantic Islands"[Mesh] OR "Senegal"[Mesh] OR "Melanesia"[Mesh] OR "Sri Lanka"[Mesh] OR "Sudan"[Mesh] OR "Swaziland"[Mesh] OR "Syria"[Mesh] OR "East Timor"[Mesh] OR "Ukraine"[Mesh] OR "Uzbekistan"[Mesh] OR "Vanuatu"[Mesh] OR "Vietnam"[Mesh] OR "Middle East"[Mesh] OR "Yemen"[Mesh] OR "Zambia"[Mesh] OR "Angola"[Mesh] OR "Albania"[Mesh] OR "Algeria"[Mesh] OR "American Samoa"[Mesh] OR "Azerbaijan"[Mesh] OR "Republic of Belarus"[Mesh] OR "Belize"[Mesh] OR "Bosnia-Herzegovina"[Mesh] OR "Botswana"[Mesh] OR "Brazil"[Mesh] OR "Bulgaria"[Mesh] OR "China"[Mesh] OR "Colombia"[Mesh] OR "Costa Rica"[Mesh] OR "Cuba"[Mesh] OR "Dominica"[Mesh] OR "Dominican Republic"[Mesh] OR "Ecuador"[Mesh] OR "Fiji"[Mesh] OR "Gabon"[Mesh] OR "Grenada"[Mesh] OR "Iran"[Mesh] OR "Iraq"[Mesh] OR "Jamaica"[Mesh] OR "Jordan"[Mesh] OR "Kazakhstan"[Mesh] OR "Lebanon"[Mesh] OR "Libya"[Mesh] OR "Macedonia (Republic)"[Mesh] OR "Malaysia"[Mesh] OR "Indian Ocean Islands"[Mesh] OR "Mexico"[Mesh] OR "Montenegro"[Mesh] OR "Namibia"[Mesh] OR "Palau"[Mesh] OR "Panama"[Mesh] OR "Peru"[Mesh] OR "Romania"[Mesh] OR "Serbia"[Mesh] OR "Seychelles"[Mesh] OR "South Africa"[Mesh] OR "Saint Lucia"[Mesh] OR "Saint Vincent and the Grenadines"[Mesh] OR "Suriname"[Mesh] OR "Thailand"[Mesh] OR "Tonga"[Mesh] OR "Tunisia"[Mesh] OR "Turkey"[Mesh] OR "Turkmenistan"[Mesh] OR "Afghanistan"[tiab] OR "Bangladesh"[tiab] OR "Benin"[tiab] OR "Burkina Faso"[tiab] OR "Burundi"[tiab] OR "Cambodia"[tiab] OR "cabo verde"[tiab] OR "Central African Republic"[tiab] OR "Chad"[tiab] OR "Comoros"[tiab] OR "Democratic Republic of the Congo"[tiab] OR "Eritrea"[tiab] OR "Ethiopia"[tiab] OR "Gambia"[tiab] OR "Guinea"[tiab] OR "Guinea-Bissau"[tiab] OR "Haiti"[tiab] OR "Kenya"[tiab] OR "Democratic People's Republic of Korea"[tiab] OR "Liberia"[tiab] OR "Madagascar"[tiab] OR "Malawi"[tiab] OR "Mali"[tiab] OR "Mozambique"[tiab] OR "Myanmar"[tiab] OR "Nepal"[tiab] OR "Niger"[tiab] OR "Rwanda"[tiab] OR "Sierra Leone"[tiab] OR "Somalia"[tiab] OR "Tajikistan"[tiab] OR "Tanzania"[tiab] OR "Togo"[tiab] OR "Uganda"[tiab] OR "Zimbabwe"[tiab] OR "Armenia"[tiab] OR "Bhutan"[tiab] OR "Bolivia"[tiab] OR "Cameroon"[tiab] OR "Cape Verde"[tiab] OR "Congo"[tiab] OR "Cote d'Ivoire"[tiab] OR "Djibouti"[tiab] OR "Egypt"[tiab] OR "El Salvador"[tiab] OR "Georgia (Republic)"[tiab] OR "Ghana"[tiab] OR "Guatemala"[tiab] OR "Guyana"[tiab] OR "Honduras"[tiab] OR "Indonesia"[tiab] OR "India"[tiab] OR “Kiribati”[tiab] OR "Kosovo"[tiab] OR "Kyrgyzstan"[tiab] OR "Kyrgyz"[tiab] OR "Laos"[tiab] OR "lao"[tiab] OR "Lesotho"[tiab] OR "Mauritania"[tiab] OR "Micronesia"[tiab] OR "Moldova"[tiab] OR "Mongolia"[tiab] OR "Morocco"[tiab] OR "Nicaragua"[tiab] OR "Nigeria"[tiab] OR "Pakistan"[tiab] OR "Papua New Guinea"[tiab] OR "Paraguay"[tiab] OR "Philippines"[tiab] OR "Independent State of Samoa"[tiab] OR "Atlantic Islands"[tiab] OR "Sao Tome"[tiab] OR Principe[tiab] OR "Senegal"[tiab] OR "Melanesia"[tiab] OR "Solomon islands"[tiab] OR "Sri Lanka"[tiab] OR "Sudan"[tiab] OR "Swaziland"[tiab] OR "Syria"[tiab] OR "East Timor"[tiab] OR "Timor leste"[tiab] OR "Ukraine"[tiab] OR "Uzbekistan"[tiab] OR "Vanuatu"[tiab] OR "Vietnam"[tiab] OR "Middle East"[tiab] OR "west bank"[tiab] OR "Gaza"[tiab] OR "Yemen"[tiab] OR "Zambia"[tiab] OR "Angola"[tiab] OR "Albania"[tiab] OR "Algeria"[tiab] OR "Samoa"[tiab] OR "Azerbaijan"[tiab] OR "Republic of Belarus"[tiab] OR "Belize"[tiab] OR "Bosnia-Herzegovina"[tiab] OR "Botswana"[tiab] OR "Brazil"[tiab] OR "Bulgaria"[tiab] OR "China"[tiab] OR "Colombia"[tiab] OR "Costa Rica"[tiab] OR "Cuba"[tiab] OR "Dominica"[tiab] OR "Dominican Republic"[tiab] OR "Ecuador"[tiab] OR "Fiji"[tiab] OR "Gabon"[tiab] OR "Grenada"[tiab] OR "Iran"[tiab] OR "Iraq"[tiab] OR "Jamaica"[tiab] OR "Jordan"[tiab] OR "Kazakhstan"[tiab] OR "Lebanon"[tiab] OR "Libya"[tiab] OR "Macedonia"[tiab] OR "Malaysia"[tiab] OR "Indian Ocean Islands"[tiab] OR "Maldives"[tiab] OR “Marshall Islands”[tiab] OR "Mauritius"[tiab] OR "Mexico"[tiab] OR "Montenegro"[tiab] OR "Namibia"[tiab] OR "Palau"[tiab] OR "Panama"[tiab] OR "Peru"[tiab] OR "Romania"[tiab] OR "Serbia"[tiab] OR "Seychelles"[tiab] OR "South Africa"[tiab] OR "Saint Lucia"[tiab] OR "Saint Vincent and the Grenadines"[tiab] OR "Suriname"[tiab] OR "Thailand"[tiab] OR "Tonga"[tiab] OR "Tunisia"[tiab] OR "Turkey"[tiab] OR "Turkmenistan"[tiab] OR "Tuvalu"[tiab] OR "low resource"[tiab] OR "under-resourced"[tiab] OR "resource poor"[tiab] OR "under-developed"[tiab] OR "underdeveloped"[tiab] OR "developing country"[tiab] OR "developing countries"[tiab] OR "developing world"[tiab] OR "third world" [tiab] OR lmic[tiab] OR (low[tiab] AND middle[tiab] AND income[tiab]) | 1,093,915 |
| 3 | #1 AND #2 | 9627 |
| 4 | randomized controlled trial[pt] OR controlled clinical trial[pt] OR randomized[tiab] OR randomised[tiab] OR randomization[tiab] OR randomisation[tiab] OR randomly[tiab] OR trial[tiab] OR Clinical trial[pt] OR “clinical trial”[tiab] OR “clinical trials”[tiab] OR "evaluation studies"[Publication Type] OR "evaluation studies as topic"[MeSH Terms] OR "evaluation study"[tiab] OR evaluation studies[tiab] OR "intervention studies"[MeSH Terms] OR "intervention study"[tiab] OR "intervention studies"[tiab] OR intervention[tiab] OR therapy[tiab] OR counsel*[tiab] OR prevention[tiab] OR preventative[tiab] OR program[tiab] OR counsel*[tiab] OR treatment[tiab] OR programme[tiab] | 6176972 |
| 5 | #3 AND #4 | 3394 |
| 6 | "Men"[Mesh] OR "Male"[Mesh] OR "Fathers"[Mesh] OR "Sex Factors"[Mesh] OR father[tiab] OR fathers[tiab] OR boys[tiab] OR men[tiab] OR man[tiab] OR male[tiab] OR gender[tiab] OR subgroup[tiab] | 7379197 |
| 7 | #5 AND #6 | 2641 |
| 8 | Family[tiab] OR Family[MeSH] OR Families[MeSH] OR “Family Members”[MeSH] OR Parenting[Mesh] OR Parent[tiab] OR "Father-Child Relations"[Mesh] OR child[tiab] OR "Child Abuse"[Mesh] OR "Domestic Violence"[Mesh] OR “child abuse”[tiab] OR marriage[tiab] OR “spousal abuse”[tiab] OR “intimate partner violence”[tiab] OR “marital satisfaction”[tiab] OR mother[tiab] OR marital[tiab] | 1199127 |
| 8 | 7 AND 8 | 579 |
| 9 | Apply Human Filter | 532 |
| 10 | English | 473 |

PSYCINFO Search December 15, 2015 at 9:00am*

| Set # | Search Terms_ PSYCINFO |  |
| --- | --- | --- |
| 1 | “Alcohol-Related Disorders” OR “Alcohol Drinking” OR “Alcoholism” OR “drinking behavior” OR problem drink* OR heavy drink* OR alcohol problem* OR risk drink* OR at-risk drink* OR alcohol depend* OR excessive drink* OR excessive alcohol* OR “alcohol consumption” OR alcohol addiction* OR risky alcohol* OR risky drink* OR “alcohol misuse” OR “alcohol abuse” OR hazardous alcohol* OR hazardous drink* OR harmful alcohol* OR harmful drink* OR ((“drinking” OR “drinkers”) AND “alcohol”) | 121,477 |
| 2 | [KEYWORD] "Developing Countries" OR "Afghanistan" OR "Bangladesh" OR "Benin" OR "Burkina Faso" OR "Burundi" OR "Cambodia" OR "Central African Republic" OR "Chad" OR "Comoros" OR "Democratic Republic of the Congo" OR "Eritrea" OR "Ethiopia" OR "Gambia" OR "Guinea" OR "Guinea-Bissau" OR "Haiti" OR "Kenya" OR "Democratic People's Republic of Korea" OR "Liberia" OR "Madagascar" OR "Malawi" OR "Mali" OR "Mozambique" OR "Myanmar" OR "Nepal" OR "Niger" OR "Rwanda" OR "Sierra Leone" OR "Somalia" OR "Tajikistan" OR "Tanzania" OR "Togo" OR "Uganda" OR "Zimbabwe" OR "Armenia" OR "Bhutan" OR "Bolivia" OR "Cameroon" OR "Cape Verde" OR "Congo" OR "Cote d'Ivoire" OR "Djibouti" OR "Egypt" OR "El Salvador" OR "Georgia (Republic)" OR "Ghana" OR "Guatemala" OR "Guyana" OR "Honduras" OR "Indonesia" OR "India" OR "Kosovo" OR "Kyrgyzstan" OR "Laos" OR "Lesotho" OR "Mauritania" OR "Micronesia" OR "Moldova" OR "Mongolia" OR "Morocco" OR "Nicaragua" OR "Nigeria" OR "Pakistan" OR "Papua New Guinea" OR "Paraguay" OR "Philippines" OR "Independent State of Samoa" OR "Atlantic Islands" OR "Senegal" OR "Melanesia" OR "Sri Lanka" OR "Sudan" OR "Swaziland" OR "Syria" OR "East Timor" OR "Ukraine" OR "Uzbekistan" OR "Vanuatu" OR "Vietnam" OR "Middle East" OR "Yemen" OR "Zambia" OR "Angola" OR "Albania" OR "Algeria" OR "American Samoa" OR "Azerbaijan" OR "Republic of Belarus" OR "Belize" OR "Bosnia-Herzegovina" OR "Botswana" OR "Brazil" OR "Bulgaria" OR "China" OR "Colombia" OR "Costa Rica" OR "Cuba" OR "Dominica" OR "Dominican Republic" OR "Ecuador" OR "Fiji" OR "Gabon" OR "Grenada" OR "Iran" OR "Iraq" OR "Jamaica" OR "Jordan" OR "Kazakhstan" OR "Lebanon" OR "Libya" OR "Macedonia (Republic)" OR "Malaysia" OR "Indian Ocean Islands" OR "Mexico" OR "Montenegro" OR "Namibia" OR "Palau" OR "Panama" OR "Peru" OR "Romania" OR "Serbia" OR "Seychelles" OR "South Africa" OR "Saint Lucia" OR "Saint Vincent and the Grenadines" OR "Suriname" OR "Thailand" OR "Tonga" OR "Tunisia" OR "Turkey" OR "Turkmenistan" OR "Afghanistan" OR "Bangladesh" OR "Benin" OR "Burkina Faso" OR "Burundi" OR "Cambodia" OR "cabo verde" OR "Central African Republic" OR "Chad" OR "Comoros" OR "Democratic Republic of the Congo" OR "Eritrea" OR "Ethiopia" OR "Gambia" OR "Guinea" OR "Guinea-Bissau" OR "Haiti" OR "Kenya" OR "Democratic People's Republic of Korea" OR "Liberia" OR "Madagascar" OR "Malawi" OR "Mali" OR "Mozambique" OR "Myanmar" OR "Nepal" OR "Niger" OR "Rwanda" OR "Sierra Leone" OR "Somalia" OR "Tajikistan" OR "Tanzania" OR "Togo" OR "Uganda" OR "Zimbabwe" OR "Armenia" OR "Bhutan" OR "Bolivia" OR "Cameroon" OR "Cape Verde" OR "Congo" OR "Cote d'Ivoire" OR "Djibouti" OR "Egypt" OR "El Salvador" OR "Georgia (Republic)" OR "Ghana" OR "Guatemala" OR "Guyana" OR "Honduras" OR "Indonesia" OR "India" OR “Kiribati” OR "Kosovo" OR "Kyrgyzstan" OR "Kyrgyz" OR "Laos" OR "lao" OR "Lesotho" OR "Mauritania" OR "Micronesia" OR "Moldova" OR "Mongolia" OR "Morocco" OR "Nicaragua" OR "Nigeria" OR "Pakistan" OR "Papua New Guinea" OR "Paraguay" OR "Philippines" OR "Independent State of Samoa" OR "Atlantic Islands" OR "Sao Tome" OR Principe OR "Senegal" OR "Melanesia" OR "Solomon islands" OR "Sri Lanka" OR "Sudan" OR "Swaziland" OR "Syria" OR "East Timor" OR "Timor leste" OR "Ukraine" OR "Uzbekistan" OR "Vanuatu" OR "Vietnam" OR "Middle East" OR "west bank" OR "Gaza" OR "Yemen" OR "Zambia" OR "Angola" OR "Albania" OR "Algeria" OR "Samoa" OR "Azerbaijan" OR "Republic of Belarus" OR "Belize" OR "Bosnia-Herzegovina" OR "Botswana" OR "Brazil" OR "Bulgaria" OR "China" OR "Colombia" OR "Costa Rica" OR "Cuba" OR "Dominica" OR "Dominican Republic" OR "Ecuador" OR "Fiji" OR "Gabon" OR "Grenada" OR "Iran" OR "Iraq" OR "Jamaica" OR "Jordan" OR "Kazakhstan" OR "Lebanon" OR "Libya" OR "Macedonia" OR "Malaysia" OR "Indian Ocean Islands" OR "Maldives" OR “Marshall Islands” OR "Mauritius" OR "Mexico" OR "Montenegro" OR "Namibia" OR "Palau" OR "Panama" OR "Peru" OR "Romania" OR "Serbia" OR "Seychelles" OR "South Africa" OR "Saint Lucia" OR "Saint Vincent and the Grenadines" OR "Suriname" OR "Thailand" OR "Tonga" OR "Tunisia" OR "Turkey" OR "Turkmenistan" OR "Tuvalu" OR "low resource" OR "under-resourced" OR "resource poor" OR "under-developed" OR "underdeveloped" OR "developing country" OR "developing countries" OR "developing world" OR "third world" OR lmic OR (low AND middle AND income) | 297,175 |
| 3 | #1 AND #2 | 9,157 |
| 5. | randomized controlled trial OR controlled clinical trial OR randomized OR randomised OR randomization OR randomisation OR “clinical trial” OR "evaluation studies" OR "evaluation studies as topic" OR "evaluation study" OR evaluation studies OR "intervention studies" OR "intervention study" OR "intervention studies" OR intervention OR therapy OR counsel* OR prevention OR preventative OR program OR counsel* OR treatment OR programme | 1,496,832 |
| 4 | #3 AND #5 | 5,501 |
| 5 | "Men" OR "Male" OR "Fathers" OR "Sex Factors" OR father OR fathers OR boys OR men OR man OR male OR gender OR subgroup | 691,956 |
| 6 | #4 AND #5 | 1948 |
| 7 | Family OR Families OR “Family Members” OR Parenting OR Parent OR "Father-Child Relations" OR child OR "Domestic Violence" OR “child abuse” OR “child maltreatment” OR marriage[tiab] OR “spousal abuse” OR “intimate partner violence” OR “marital satisfaction” OR mother OR marital OR “Parent-Child” OR “parent-child relationship” | 943,467 |
| 8 | 6 & 7 | 631 |
| 9 | English Filter | 525 |

WEB OF SCIENCE December 9, 2015 3:45pm*

| Set # | Search Terms_ WEB OF SCIENCE |  |
| --- | --- | --- |
| 1 | “Alcohol-Related Disorders” OR “Alcohol Drinking” OR “Alcoholism” OR “drinking behavior” OR problem drink* OR heavy drink* OR alcohol problem* OR risk drink* OR at-risk drink* OR alcohol depend* OR excessive drink* OR excessive alcohol* OR “alcohol consumption” OR alcohol addiction* OR risky alcohol* OR risky drink* OR “alcohol misuse” OR “alcohol abuse” OR hazardous alcohol* OR hazardous drink* OR harmful alcohol* OR harmful drink* OR ((“drinking” OR “drinkers”) AND “alcohol”) | 177,543 |
| 2 | "Developing Countries" OR "Afghanistan" OR "Bangladesh" OR "Benin" OR "Burkina Faso" OR "Burundi" OR "Cambodia" OR "Central African Republic" OR "Chad" OR "Comoros" OR "Democratic Republic of the Congo" OR "Eritrea" OR "Ethiopia" OR "Gambia" OR "Guinea" OR "Guinea-Bissau" OR "Haiti" OR "Kenya" OR "Democratic People's Republic of Korea" OR "Liberia" OR "Madagascar" OR "Malawi" OR "Mali" OR "Mozambique" OR "Myanmar" OR "Nepal" OR "Niger" OR "Rwanda" OR "Sierra Leone" OR "Somalia" OR "Tajikistan" OR "Tanzania" OR "Togo" OR "Uganda" OR "Zimbabwe" OR "Armenia" OR "Bhutan" OR "Bolivia" OR "Cameroon" OR "Cape Verde" OR "Congo" OR "Cote d'Ivoire" OR "Djibouti" OR "Egypt" OR "El Salvador" OR "Georgia (Republic)" OR "Ghana" OR "Guatemala" OR "Guyana" OR "Honduras" OR "Indonesia" OR "India" OR "Kosovo" OR "Kyrgyzstan" OR "Laos" OR "Lesotho" OR "Mauritania" OR "Micronesia" OR "Moldova" OR "Mongolia" OR "Morocco" OR "Nicaragua" OR "Nigeria" OR "Pakistan" OR "Papua New Guinea" OR "Paraguay" OR "Philippines" OR "Independent State of Samoa" OR "Atlantic Islands" OR "Senegal" OR "Melanesia" OR "Sri Lanka" OR "Sudan" OR "Swaziland" OR "Syria" OR "East Timor" OR "Ukraine" OR "Uzbekistan" OR "Vanuatu" OR "Vietnam" OR "Middle East" OR "Yemen" OR "Zambia" OR "Angola" OR "Albania" OR "Algeria" OR "American Samoa" OR "Azerbaijan" OR "Republic of Belarus" OR "Belize" OR "Bosnia-Herzegovina" OR "Botswana" OR "Brazil" OR "Bulgaria" OR "China" OR "Colombia" OR "Costa Rica" OR "Cuba" OR "Dominica" OR "Dominican Republic" OR "Ecuador" OR "Fiji" OR "Gabon" OR "Grenada" OR "Iran" OR "Iraq" OR "Jamaica" OR "Jordan" OR "Kazakhstan" OR "Lebanon" OR "Libya" OR "Macedonia (Republic)" OR "Malaysia" OR "Indian Ocean Islands" OR "Mexico" OR "Montenegro" OR "Namibia" OR "Palau" OR "Panama" OR "Peru" OR "Romania" OR "Serbia" OR "Seychelles" OR "South Africa" OR "Saint Lucia" OR "Saint Vincent and the Grenadines" OR "Suriname" OR "Thailand" OR "Tonga" OR "Tunisia" OR "Turkey" OR "Turkmenistan" OR "Afghanistan" OR "Bangladesh" OR "Benin" OR "Burkina Faso" OR "Burundi" OR "Cambodia" OR "cabo verde" OR "Central African Republic" OR "Chad" OR "Comoros" OR "Democratic Republic of the Congo" OR "Eritrea" OR "Ethiopia" OR "Gambia" OR "Guinea" OR "Guinea-Bissau" OR "Haiti" OR "Kenya" OR "Democratic People's Republic of Korea" OR "Liberia" OR "Madagascar" OR "Malawi" OR "Mali" OR "Mozambique" OR "Myanmar" OR "Nepal" OR "Niger" OR "Rwanda" OR "Sierra Leone" OR "Somalia" OR "Tajikistan" OR "Tanzania" OR "Togo" OR "Uganda" OR "Zimbabwe" OR "Armenia" OR "Bhutan" OR "Bolivia" OR "Cameroon" OR "Cape Verde" OR "Congo" OR "Cote d'Ivoire" OR "Djibouti" OR "Egypt" OR "El Salvador" OR "Georgia (Republic)" OR "Ghana" OR "Guatemala" OR "Guyana" OR "Honduras" OR "Indonesia" OR "India" OR “Kiribati” OR "Kosovo" OR "Kyrgyzstan” OR "Kyrgyz" OR "Laos" OR "lao" OR "Lesotho" OR "Mauritania" OR "Micronesia" OR "Moldova" OR "Mongolia" OR "Morocco" OR "Nicaragua" OR "Nigeria" OR "Pakistan" OR "Papua New Guinea" OR "Paraguay" OR "Philippines" OR "Independent State of Samoa" OR "Atlantic Islands" OR "Sao Tome" OR Principe OR "Senegal" OR "Melanesia" OR "Solomon islands" OR "Sri Lanka" OR "Sudan" OR "Swaziland" OR "Syria" OR "East Timor" OR "Timor leste" OR "Ukraine" OR "Uzbekistan" OR "Vanuatu" OR "Vietnam" OR "Middle East" OR "west bank" OR "Gaza" OR "Yemen" OR "Zambia" OR "Angola" OR "Albania" OR "Algeria" OR "Samoa" OR "Azerbaijan" OR "Republic of Belarus" OR "Belize" OR "Bosnia-Herzegovina" OR "Botswana" OR "Brazil" OR "Bulgaria" OR "China" OR "Colombia" OR "Costa Rica" OR "Cuba" OR "Dominica" OR "Dominican Republic" OR "Ecuador" OR "Fiji" OR "Gabon" OR "Grenada" OR "Iran" OR "Iraq" OR "Jamaica" OR "Jordan" OR "Kazakhstan" OR "Lebanon" OR "Libya" OR "Macedonia" OR "Malaysia" OR "Indian Ocean Islands" OR "Maldives" OR “Marshall Islands” OR "Mauritius" OR "Mexico" OR "Montenegro" OR "Namibia" OR "Palau" OR "Panama" OR "Peru" OR "Romania" OR "Serbia" OR "Seychelles" OR "South Africa" OR "Saint Lucia" OR "Saint Vincent and the Grenadines" OR "Suriname" OR "Thailand" OR "Tonga" OR "Tunisia" OR "Turkey" OR "Turkmenistan" OR "Tuvalu" OR "low resource" OR "under-resourced" OR "resource poor" OR "under-developed" OR "underdeveloped" OR "developing country" OR "developing countries" OR "developing world" OR "third world" OR lmic OR (low AND middle AND income) | 1,975,846 |
| 3 | #1 AND #2 | [12,799](http://apps.webofknowledge.com.proxy.lib.duke.edu/summary.do?product=WOS&doc=1&qid=3&SID=1CrBFHBHhVfMDdHTTuA&search_mode=CombineSearches&update_back2search_link_param=yes) |
| 5. | randomized controlled trial OR controlled clinical trial OR randomized OR randomised OR randomization OR randomisation OR “clinical trial” OR "evaluation studies" OR "evaluation studies as topic" OR "evaluation study" OR evaluation studies OR "intervention studies" OR "intervention study" OR "intervention studies" OR intervention OR therapy OR counsel* OR prevention OR preventative OR program OR counsel* OR treatment OR programme | 6,827,281 |
| 4 | #3 AND #5 | 5268 |
| 5 | "Men" OR "Male" OR "Fathers" OR "Sex Factors" OR father OR fathers OR boys OR men OR man OR male OR gender OR subgroup | 1,976,964 |
| 6 | #4 AND #5 | 1981 |
| 7 | Family OR Families OR “Family Members” OR Parenting OR Parent OR "Father-Child Relations" OR child OR "Domestic Violence" OR “child abuse” OR “child maltreatment” OR marriage[tiab] OR “spousal abuse” OR “intimate partner violence” OR “marital satisfaction” OR mother OR marital OR “Parent-Child” OR “parent-child relationship” | 2,431,663 |
| 8 | 6 & 7 | 646 |
| 9 | English (561) | 561 |

*****This search using the same terms and order was updated December 2016 resulting in 1 included article.
